# Uterine torsion in twin pregnancy with complete bicorporeal uterus, double cervix, longitudinal non-obstructing vaginal septum - a case report

**DOI:** 10.3389/fsurg.2023.1082955

**Published:** 2023-03-23

**Authors:** Qianqian Gao, Jinqiu Xiong, Yuchun Zhu

**Affiliations:** ^1^ Departments of Obstetrics, Weifang People’s Hospital, Weifang, China; ^2^ Departments of General Surgery, Weifang People’s Hospital, Weifang, China

**Keywords:** uterine torsion, twin pregnancy, case report, surgery, complete bicorporeal uterus

## Abstract

Even though uterine torsion is a rare obstetric complication in humans, it has been linked to severe complications like placental abruption, uterine rupture, foetal bradycardia, or even death. Here, we present a rare case of maternal shortening and prolonged foetal bradycardia. The patient suffered from congenital malformations of the female genital tract, which were classified as a complete bicorporeal uterus, a double “normal” cervix, and a longitudinal non-obstructing vaginal septum (U3b/C2/V1). The patient had an emergency caesarean section due to suspected placental abruption. Uterine torsion was found during the surgery, and the postoperative recovery was good. Obstetricians should be aware of the possibility of uterine torsion as a complication of pregnancy to avoid a delayed diagnosis of uterine torsion, especially in patients with genital malformations. During the surgery, there could be serious damage to blood vessels and tissues around the uterus due to an unclear surgical field, and difficulties in exposing the uterine body should be considered.

## 1. Introduction

Uterine torsion, which is defined as a rotation of >45° around the long axis, is a rare obstetric complication in humans that can lead to severe complications if ignored. Twin pregnancy, with simultaneous pregnancy in each uterus and a complete bicorporeal uterus, is another rare and complex clinical situation. To date, most studies in the literature have been case reports of single pregnancies, and we present a case of gravid uterus torsion in a complete bicorporeal uterus with a twin pregnancy during the third trimester.

## 2. Patient and case presentation

A 33-year-old para-2 mother was hospitalised at 33 weeks of gestation because of a threatened premature delivery. The patient had a complete bicorporeal uterus. She had a healthy full-term baby through an uncomplicated lower right uterine segment caesarean section prior to this pregnancy. The patient conceived spontaneously. Two foetuses from a twin pregnancy (cephalic position and transverse lie) were discovered in the bilateral uterine cavities. She had no significant disease history and denied a history of smoking.

The patient's vital signs were stable at admission. She denied vaginal bleeding and described normal foetal movement before presentation, except for irregular uterine contractions. Laboratory examination showed normal results, and the patient suddenly experienced dizziness and fatigue after rolling over. Physical examination revealed hypotension (blood pressure, 81/55 mmHg) and tenderness in the right lower abdomen. The foetal heart rate (left/right) was 130/60 beats per minute. An emergency caesarean section was rapidly performed under spinal anaesthesia due to maternal shock and foetal bradycardia. We considered placental abruption or uterine rupture as a preoperative diagnosis, and no imaging examination was performed due to the urgency of the event. The abdomen was opened using a Pfannenstiel incision. After opening the peritoneum, we examined one uterus and did a caesarean section on its lower part. The first neonate was delivered without difficulty, with an Apgar score of 10 points (1/5/10 min). The other uterus was congested, and the peritoneum of the uterus and bladder were invisible. No obvious rupture of the uterus or difficult exposure of the ipsilateral fallopian tube and ovary were observed during exploration. An engorged venous plexus crossing the anterior operating field was observed. Another neonate was delivered with an Apgar score of 10 (1/5/10 min). Due to the urgency of the situation, it took only 21 min from the discovery of the abnormality to the delivery of the foetus by caesarean section.

**Figure 1:**
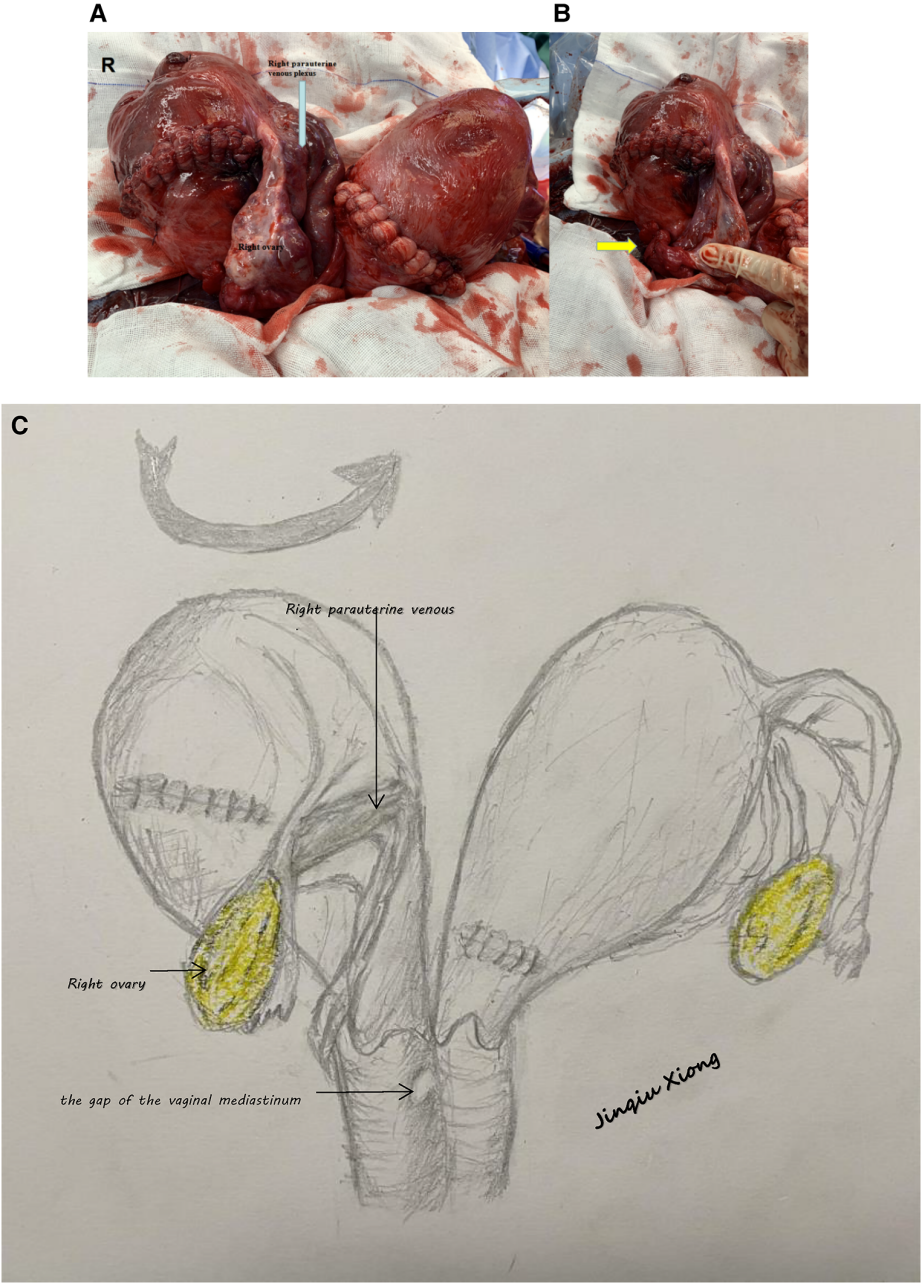
(**A**) Uterus after foetal delivery (Uterus exteriorized intraoperatively). (**B**) Torsion of the cervix and uterine body (arrow; the peritoneum of the uterus and bladder was invisible). (**C**) Schematic figure of uterine torsion.

**Figure 2:**
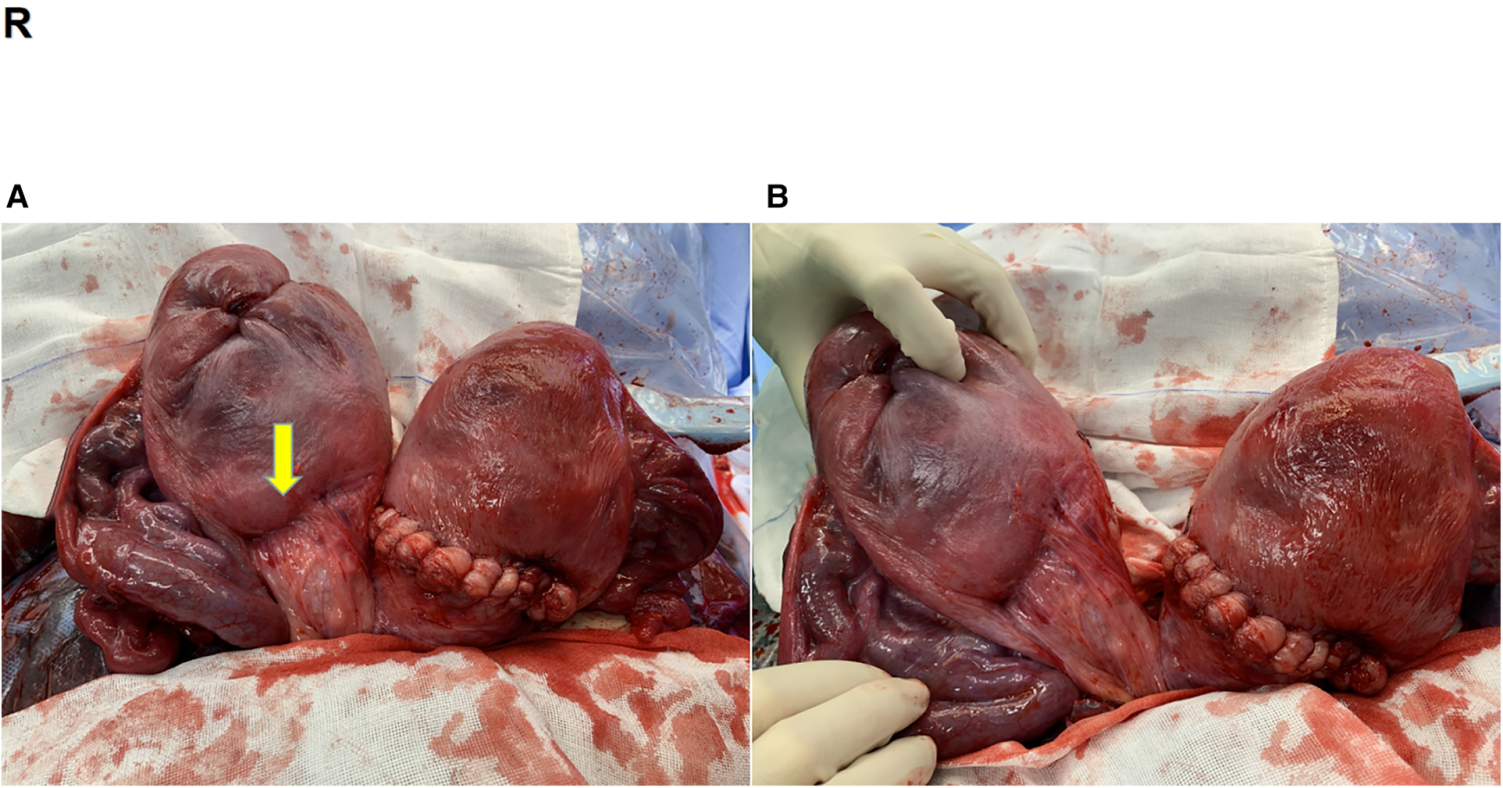
(**A**) Uterus after restoration (arrow; previous surgical scar in the lower right uterine segment). (**B**) Biuterine malformation.

**Figure 3:**
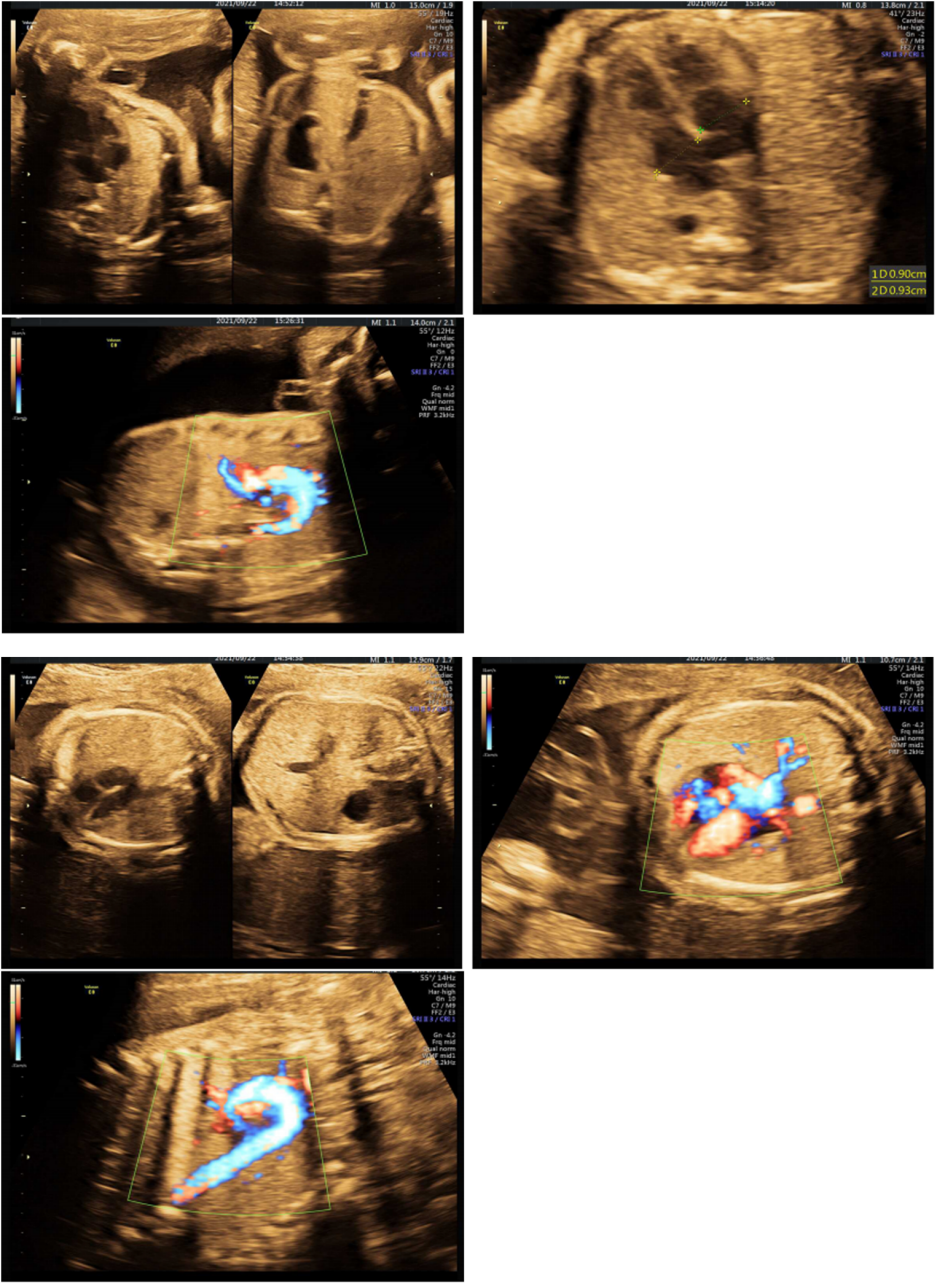
Doppler echocardiography of two fetuses.

After the newborns and placentas were delivered, the right uterus was rotated 180° anticlockwise at the junction of the cervix and uterine corpus along the long axis of the uterus. This caused hyperaemia and distension of the parauterine venous plexus (Figure 1). The rotation was corrected manually without difficulty, and we performed a posterior transverse caesarean delivery (Figure 2). The colour of the uterine muscle layer rapidly returned to normal after restoration, and no signs of placental abruption were observed. During the postoperative physical examination, we discovered the vaginal mediastinum and double cervix, and the gap of the mediastinum, which was 2 cm away from the cervix, connected the two vaginas, which were classified as a complete bicorporeal uterus, a double “normal” cervix, and a longitudinal non-obstructing vaginal septum (U3b/C2/V1) according to the European Society of Human Reproduction and Embryology and the European Society for Gynaecological Endoscopy Classification (1), and was classified as uterus didelphys with a complete longitudinal vaginal septum according to the American Society for Reproductive Medicine 2021 classification (2). The estimated blood loss volume was 600 ml. Oxytocin and prophylactic antibiotic were administered. Low-molecular-weight heparin was administered postoperatively to prevent thrombosis due to preoperative uterine ischaemia and reperfusion. The patient recovered well and was discharged on the fifth postoperative day. Doppler echocardiography of the two foetuses was normal (Figure 3). Two neonates underwent Doppler echocardiography after birth, and one had patent ductus arteriosus and patent foramen ovale. Two neonates were followed up, and so far, they have been in good condition.

## 3. Discussion

The enlarged uterine body is usually dextral (<45°) during pregnancy due to the presence of the rectum sigmoid colon, which is a physiological change. Females with congenital uterine anomalies may be at increased risk of spontaneous abortion, premature birth, foetal growth restriction, foetal malpresentation, and abnormal placental insertion (3, 4). The aetiology of uterine torsion is unknown, but the uterus can twist because of hysteromyoma (5, 6), uterine malformation (7), external cephalic version (8, 9), pelvic malformation, loosely suspended uterus, malpresentation (10), pelvic adhesions, which may cause placental abruption, placental perfusion insufficiency (11), foetal distress (8), foetal death (12), intrauterine growth restriction (13), maternal shock, and other fatal complications. However, few cases have occurred without discernible causes (14). Symptoms of uterine torsion vary depending on the degree and duration of torsion. Due to its rarity and atypical clinical symptoms, uterine torsion is usually an acute abdominal condition that is difficult to diagnose preoperatively. Only rare asymptomatic cases during pregnancy have been diagnosed intraoperatively (15). Lower abdominal pain was the most common symptom. By comparing placental location, ultrasonography can aid in diagnosis. Differential diagnoses include placental abruption, uterine rupture, premature labour, appendicitis, acute pyelonephritis, urinary obstruction, hematoma of the broad ligament of the uterus, etc.

Congenital malformations of the female genital tract are common benign conditions that are associated with reproductive problems depending on the type and degree of anatomical distortion, and in our case, when there is an abnormal foetal presentation and uterine malformation exists, sudden postural change may be the precipitating factor.We have used the CARE criteria (https://www.care-statement.org/checklist) to describe the case report. We have submitted as many details about the patient as possible in the hopes of providing some instructive information. Unfortunately, no image of a patient's gynaecological examination or cadiotocography registration of the foetal heart is available.

Obstetricians should be aware of this complication to avoid a delayed diagnosis of uterine torsion. Prompt surgical treatment should be performed to avoid a hysterectomy for uterine necrosis. During the surgery, there could be serious damage to the blood vessels and tissues around the uterus because of an unclear surgical field, and the difficulties in exposing the uterine body should be considered.

## Data Availability

The original contributions presented in the study are included in the article/Supplementary Material, further inquiries can be directed to the corresponding author.
